# Supplementary material for: Deficiency of ChPks and ChThr1 Inhibited DHN-Melanin Biosynthesis, Disrupted Cell Wall Integrity and Attenuated Pathogenicity in Colletotrichum higginsianum
Source: Int J Mol Sci. 2023 Nov 2;24(21):15890. doi: 10.3390/ijms242115890 (PMC10650501; doi:10.3390/ijms242115890)
Supplement: Supplementary file 1 [file ijms-24-15890-s001.zip › ijms-2660389-supplementary.pdf]

Supplementary Materials

Deficiency of ChPks and ChThr1 Inhibited DHN-Melanin Biosynthesis, Disrupted Cell Wall Integrity and Attenuated Pathogenicity in *Colletotrichum higginsianum*

Lingtao Duan †, Li Wang †, Weilun Chen, Zhenrui He, Erxun Zhou * and Yiming Zhu *

Guangdong Province Key Laboratory of Microbial Signals and Disease Control, College of Plant Protection, South China Agricultural University, Guangzhou 510642, China

* Correspondence: Correspondence: exzhou@scau.edu.cn (E.Z.); zhu_yiming1992@scau.edu.cn (Y.Z.); Tel./Fax: +86-20-8528-6089 (E.Z.)

† These authors contributed equally to this work.

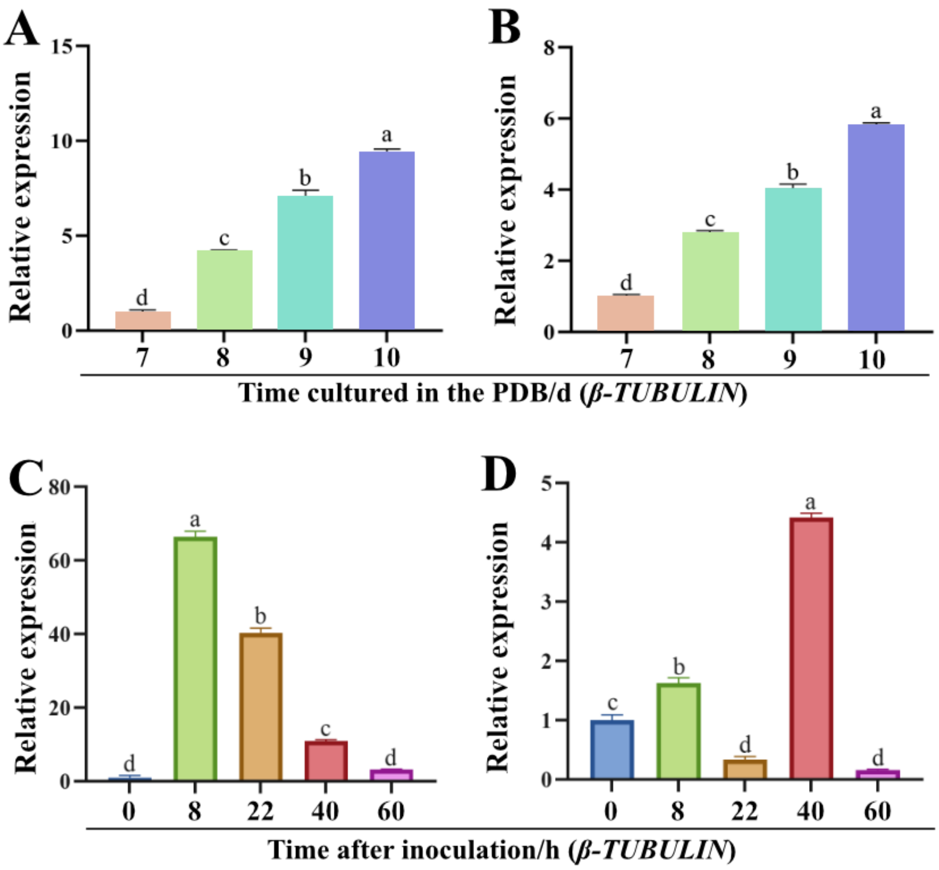

Figure S1. Expression analyses of genes *ChPKS* and *ChTHR1* during hypha and appressorium melanization of *Colletotrichum higginsianum*. (**A**,**B**) Expression patterns of genes *ChPKS* and *ChTHR1* during hypha melanization with *β-TUBULIN* as the endogenous reference gene. (**C**,**D**) Expression patterns of genes *ChPKS* and *ChTHR1* during appressorium melanization with *β-TUBULIN* as the endogenous reference gene. Error bars represent standard deviations from three replicates, experimental data were analyzed by One-way ANOVA. Different letters indicate a significant difference at $p < 0.05$.

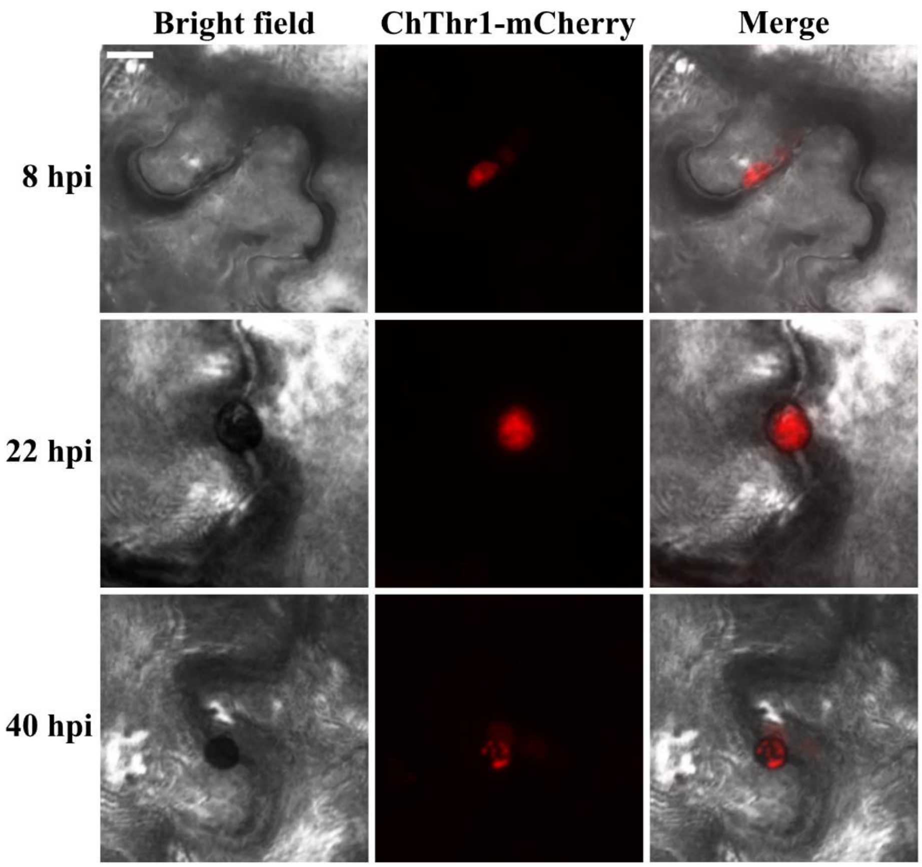

Figure S2. Fluorescence microscopy images of the ChThr1-mCherry during infection of *Colletotrichum higginsianum*. Images were analyzed by confocal microscopy to detect the mCherry signal at different time points during infection. Scale bar = 10 μm.

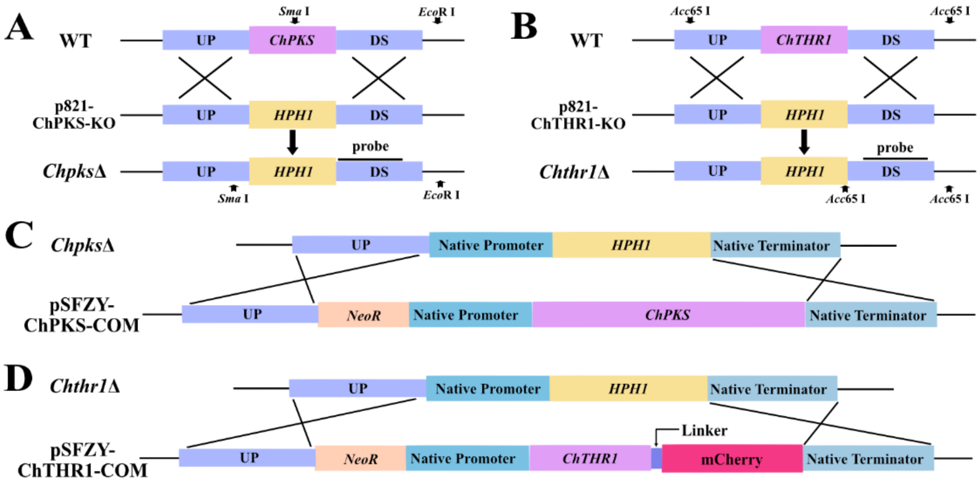

Figure S3. Targeted gene knockout and complementation of *ChPKS* and *ChTHR1* in *Colletotrichum higginsianum*. (**A**,**B**) Schematic map illustrating the construction of gene knockout vectors for genes *ChPKS* and *ChTHR1*, including restriction enzyme sites within the genomic region of interest. A bold line below the disruption construct represents the sequence used as a probe in Southern blot analysis. (**C**,**D**) Schematic map illustrating the construction of gene complementation vectors for genes *ChPKS* and *ChTHR1*.

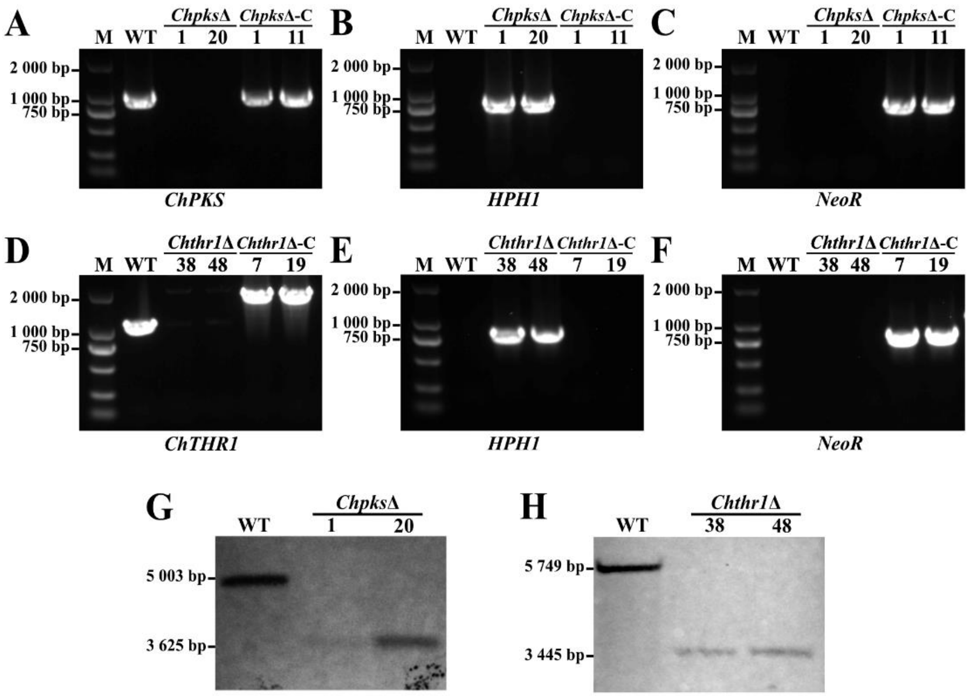

Figure S4. PCR and Southern blotting validation of the *Chpks*Δ and *Chthr1*Δ mutants of *Colletotrichum higginsianum*. (**A**–**C**) PCR products were amplified from WT, *Chpks*Δ and *Chpks*Δ-C strains using primer pairs PKS-F/R, HPH1-F/R and NeoR-F/R. (**D**–**F**) PCR products were amplified from WT, *Chthr1*Δ and *Chthr1*Δ-C strains using primer pairs PKS-F/R, HPH1-F/R and NeoR-F/R. (**G**) Southern blotting analysis of the WT and *Chpks*Δ strains. (**H**) Southern blotting analysis of the WT and *Chthr1*Δ strains.

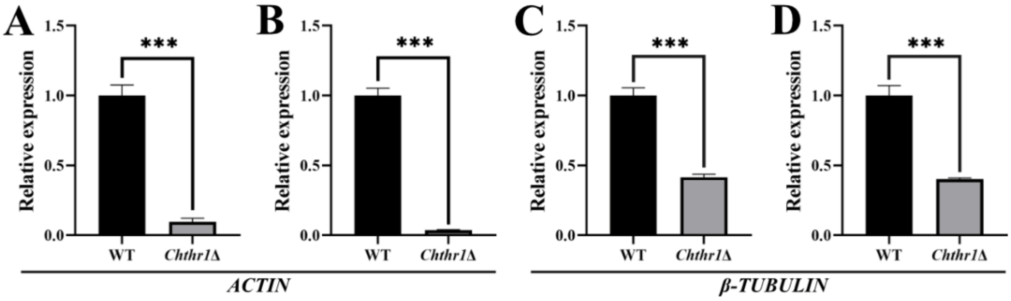

Figure S5. Expression analysis of gene *ChTHNR* of *Colletotrichum higginsianum* at 4 d (**A**,**C**) and 7 d (**B**,**D**) of culture in the *Chthr1*Δ mutant. Error bars represent standard deviations from three replicates. The experimental data were analyzed by One-way ANOVA. *** indicate $p < 0.001$.
